# Urinary organic acid levels and their associations with clinical characteristics in patients with schizophrenia

**DOI:** 10.1007/s11306-026-02479-5

**Published:** 2026-06-16

**Authors:** Yavuz Yılmaz, Halef Okan Doğan, Ayşegül Murat, Gökmen Zararsız

**Affiliations:** 1 Department of Psychiatry, Faculty of Medicine, Sivas Cumhuriyet University, Sivas, Turkey; 2 Department of Medical Biochemistry, Faculty of Medicine, Sivas Cumhuriyet University, Sivas, Turkey; 3 Department of Biostatistics, Erciyes University School of Medicine, Kayseri, Türkiye; 4 Erciyes Teknopark, Hematainer Biotechnology and Health Products Inc, Kayseri, Türkiye

**Keywords:** Schizophrenia, Urinary organic acids, Metabolomics, Propanoate metabolism, Intermediary metabolism

## Abstract

**Introduction:**

Schizophrenia is a chronic psychiatric disorder characterized by substantial biological and clinical heterogeneity. Beyond classical neurotransmitter-based models, increasing evidence suggests that systemic metabolic alterations may contribute to its pathophysiology.

**Objectives:**

This study aimed to characterize urinary organic acid profiles in patients with schizophrenia and investigate their associations with clinical characteristics and pathway-level metabolic alterations.

**Methods:**

In this cross-sectional study, urinary organic acids were quantified using liquid chromatography–tandem mass spectrometry (LC–MS/MS) in 55 patients with schizophrenia and 30 age- and sex-matched healthy controls. Organic acid concentrations were normalized to urinary creatinine levels. Clinical severity was evaluated using the Positive and Negative Syndrome Scale and the Clinical Global Impressions–Severity scale. Differential metabolite analysis, subgroup comparisons, principal component analysis, correlation analyses, and pathway enrichment analyses were performed.

**Results:**

Patients with schizophrenia demonstrated widespread alterations in urinary organic acid profiles compared with healthy controls, with 40 metabolites remaining significantly different after false discovery rate correction. Subgroup analyses identified additional metabolomic variation according to symptom severity, treatment adherence, family history, and current treatment status. Principal component analysis demonstrated partial separation between patients and controls, whereas subgroup distributions showed substantial overlap. Correlation analyses revealed predominantly weak-to-moderate associations between clinical variables and urinary metabolite concentrations. Pathway enrichment analysis identified propanoate metabolism as the only pathway that remained statistically significant after multiple testing correction, while several additional pathways demonstrated nominal enrichment.

**Conclusion:**

These findings suggest that schizophrenia is associated with broad alterations in urinary metabolomic profiles and support the possibility that intermediary metabolic pathways may contribute to the biological complexity and heterogeneity of the disorder. Further longitudinal and validation studies are needed to clarify the biological and clinical relevance of these observations.

**Supplementary Information:**

The online version contains supplementary material available at https://doi.org/10.1007/s11306-026-02479-5.

## Introduction

Schizophrenia is a chronic and disabling psychiatric disorder characterized by marked heterogeneity in clinical presentation, illness course, and treatment response. Despite extensive research, the underlying neurobiological mechanisms remain only partially understood, and conventional neurotransmitter-based models do not fully explain the biological complexity of the disorder.

Recent evidence suggests that schizophrenia may involve broader systemic disturbances extending beyond neurotransmission, particularly in metabolic regulation and cellular bioenergetics. Alterations in mitochondrial function, oxidative stress, energy homeostasis, and associated metabolic abnormalities have all been implicated in the disorder (Prabakaran et al., 2004; Iwamoto et al., 2005; Martins-de-Souza et al., 2009; Roberts, 2021; Henkel et al., 2022; Purcell et al., 2023). Emerging metabolomic studies further indicate that these metabolic changes may not be restricted to isolated biochemical pathways but instead reflect interconnected alterations across intermediary metabolism and systemic biological processes (Cai et al., 2012).

Metabolic alterations in schizophrenia extend beyond classical energy-related pathways. Short-chain fatty acids (SCFAs), particularly butyrate produced by gut microbiota, have received increasing attention because of their roles in neurodevelopment, neuroimmune regulation, blood–brain barrier integrity, and epigenetic signaling (Cryan et al., 2019; Dalile et al., 2019; Bourassa et al., 2016). Studies examining the microbiota–gut–brain axis have demonstrated significant differences in gut microbial composition in schizophrenia, suggesting that microbiota-associated metabolic pathways may contribute to neurobiological processes relevant to psychopathology (Li et al., 2021; Murray et al., 2023; Szeligowski et al., 2020).

Another important dimension of metabolic dysregulation in schizophrenia relates to lipid metabolism and treatment-associated metabolic changes. Antipsychotic treatment has been shown to influence lipid utilization, alter metabolic flexibility, and contribute to systemic metabolic disturbances including dyslipidemia (Albaugh et al., 2012; de Leon et al., 2008; Li et al., 2020; Yan et al., 2013). These findings suggest that metabolic alterations observed in schizophrenia may arise from both illness-related and treatment-associated mechanisms.

Another emerging area of interest is propanoate metabolism and branched-chain amino acid (BCAA) metabolism. Severe disturbances in these pathways characterize inherited metabolic disorders such as methylmalonic and propionic acidemia (Deodato et al., 2006; Head et al., 2022; Huemer et al., 2017; Longo et al., 2022), whereas milder alterations in intermediary metabolic pathways have increasingly been proposed to contribute to complex neuropsychiatric phenotypes. BCAA metabolism contributes to broader intermediary metabolism and supports systemic metabolic regulation (Mochel, 2017; Sonnewald, 2014).

Taken together, these findings suggest that metabolic dysregulation may represent an important biological dimension of schizophrenia. However, studies systematically evaluating urinary organic acid profiles and their relationship with pathway-level metabolic alterations and clinical heterogeneity remain limited. Therefore, the present study aimed to characterize urinary organic acid profiles in individuals with schizophrenia, compare them with healthy controls, and investigate their associations with clinical characteristics including symptom severity, treatment-related factors, and family history. In addition, pathway enrichment and multivariate analyses were performed to explore broader metabolic patterns underlying the observed metabolomic alterations. Improving our understanding of these metabolic alterations may provide further insight into the systemic biology of schizophrenia.

## Methods

### Participants

This study included 55 patients who were diagnosed with schizophrenia according to DSM-5 diagnostic criteria and were under follow-up at the Department of Psychiatry, Faculty of Medicine, Sivas Cumhuriyet University. The control group consisted of 30 healthy volunteers without any chronic illness, malignancy, autoimmune disease, or systemic infection, and who were matched to the patient group in terms of age and gender. Assessments of both the patient and control groups were conducted at the outpatient clinic of the Department of Psychiatry. Sociodemographic data were collected using a structured form developed by researchers. Additionally, the Positive and Negative Syndrome Scale (PANSS) and the Clinical Global Impressions Scale (CGI) were administered to the patient group. None of the patients were taking any medications other than psychotropic drugs, and they were not subject to any specific dietary restrictions.

### Measures

#### Positive and negative syndrome scale (PANSS)

The PANSS is a clinician-rated, Likert-type scale designed to assess positive and negative symptoms, as well as general psychopathology, in individuals with schizophrenia. It consists of a total of 30 items, including 7 items for positive symptoms, 7 items for negative symptoms, and 16 items for general psychopathology. The scale was developed by Kay et al. (1987), and the reliability and validity of the Turkish version have been established (Kostakoğlu et al., 1999).

#### Clinical global impressions scale (CGI)

The CGI was developed by Guy (1976) to evaluate the clinical course of psychiatric disorders. It is a three-dimensional scale applicable to patients of all ages (Forkmann et al., 2011). The first component, CGI-Severity, assesses the severity of illness at the time of evaluation on a 7-point scale ranging from 1 (Normal, not at all ill) to 7 (Among the most extremely ill patients). The second component, CGI-Improvement, measures changes in the patient’s condition compared to the baseline, also on a 7-point scale from 1 (Very much improved) to 7 (Very much worse). In this study, the severity subscale was administered during the initial inpatient interview, and the improvement subscale was administered at discharge. Patients diagnosed with affective psychosis were compared with other diagnostic groups in terms of initial illness severity and clinical improvement at discharge. The third component, CGI–Side Effects, which is rated on a 4-point scale, was not utilized in this study.

### Biochemical analyses

#### Sample

Spot urine samples were collected from participants after 8–10 h of overnight fasting using sterile, preservative-free urine containers. All samples were obtained in the morning (first void or midstream not specified) and immediately transported on ice to the laboratory, where they were aliquoted and stored at − 80 °C until analysis to minimize degradation of metabolites.

#### Urine creatinine measurement

Prior to analysis, urine samples were centrifuged at 3000 rpm for 10 min at 4 °C to remove debris. The supernatant was used to determine creatinine concentrations using the kinetic Jaffe method on a Roche Cobas c-702 analyzer (Roche Diagnostics, Mannheim, Germany). Urinary organic acid concentrations were normalized to urinary creatinine levels and expressed as µmol analyte per mmol creatinine.

#### Sample preparation and LC-MS/MS analysis

Urinary organic acids were analyzed using a liquid chromatography–tandem mass spectrometry (LC–MS/MS) method on an Agilent 1290 Infinity HPLC platform (Agilent Technologies, Santa Clara, CA, USA) coupled to an Agilent 6470 triple quadrupole mass spectrometer equipped with an Agilent Jet Stream (AJS) electrospray ionization (ESI) source. The HPLC system consisted of a binary pump (model G4220A), thermostated column compartment (G1316C), and autosampler (G7167B). All analyses were carried out using the CE-IVD certified Jasem Organic Acids LC-MS/MS Analysis Kit (Altium Laboratuvar Cihazları Pazarlama San. ve Tic. Inc., Istanbul, Turkey), which provides reagents, calibration standards, and analytical specifications optimized for the quantitative determination of urinary organic acids.

Sample preparation was performed according to the manufacturer’s instructions. In brief, urine samples were first diluted 1:10 (v/v) with the kit’s dilution reagent (Reagent 1). Subsequently, 100 µL of the diluted sample (or calibrator) was transferred to a sample vial, followed by the addition of 50 µL of the stable isotope-labeled internal standard (IS) mixture supplied with the kit. The mixture was vortexed for 5 s at room temperature, after which 850 µL of the dilution reagent was added. Samples were mixed briefly (5 s) before being loaded into the LC-MS/MS system.

The chromatographic separation was achieved on the dedicated analytical column included in the kit, using the provided mobile phases A and B with the gradient conditions defined in the kit protocol. Detection was performed using both positive and negative electrospray ionization modes in multiple-reaction monitoring (MRM) mode. The instrument was calibrated using six-point external calibration curves prepared from the supplied calibrators. Internal standard–based quantitation was employed to correct for matrix effects and variability in ionization efficiency.

Data were acquired and processed with Agilent MassHunter software packages (Acquisition v10.1, Qualitative Analysis v10.0, and Quantitative Analysis v10.0). The analytical performance of the method was consistent with the kit specifications: linearity for all targeted analytes was observed over the range of 0.1–100 µg/mL with coefficients of determination (R²) exceeding 0.990. The method accuracy across the tested analytes ranged from 73.1% to 120.0%, and the intra-assay relative standard deviation (RSD) did not exceed 17.1%.

### Statistical analysis and visualization

All urinary organic acid levels were normalized to urinary creatinine concentrations and expressed as µmol/mmol creatinine to account for urine concentration variability. Unless otherwise stated, all metabolomic analyses and visualizations were performed in JupyterLab (v4.4.9) using the Python (v3.11.0) programming language (Ernesti & Kaiser, 2025; Kluyver et al., 2016). The analyses were performed using relevant Python libraries, primarily Pandas (v2.3.3) and NumPy (v1.26.4), and default parameters were preferred unless otherwise specified (Ernesti & Kaiser, 2025; McKinney, 2015; Harris et al., 2020). Normalized metabolite data were scaled using the StandardScaler method in the scikit-learn (v1.7.2) library for data standardization purposes, and then evaluated using principal component analysis (PCA, n_component = 2) (Pedregosa et al., 2011).

The normality of continuous variable distributions was assessed using the Shapiro–Wilk test. The SciPy (v1.16.2) Mann–Whitney U test was used for comparisons of continuous variables between two groups, and the Chi-square test was used for the analysis of categorical variables (Virtanen et al., 2020). Multiple testing corrections were performed using the Benjamini–Hochberg false discovery rate (FDR) method with the Statsmodels (v0.14.5) library, and an adjusted p-value (p-adj/FDR) < 0.05 was used as a statistical threshold (Benjamini & Hochberg, 1995). In analyses where statistical significance could not be achieved after FDR correction, a *p* < 0.10 threshold was used to evaluate differences at the trend level.

Treatment-related subgroup analyses were additionally performed to evaluate potential treatment-associated metabolic variation. Patients were categorized according to current treatment status (antipsychotic treatment only vs. antipsychotic plus mood stabilizer treatment), and comparisons were conducted using the same statistical framework applied to other clinical subgroup analyses.

The Matplotlib (v3.10.9) library was primarily used in creating the graphs; the Python math and textwrap modules were also used for editing and formatting (Tosi, 2009). Venn (v0.1.3) diagrams and UpSetPlot (v0.9.0) visualizations were used to evaluate the overlap among metabolites identified as significant across six comparisons conducted within the scope of the study (LankyCyril, n.d.; Lex et al., 2014). The combinations function from the Python itertools module was used to identify common metabolite combinations. Associations between organic acid concentrations and clinical parameters were evaluated using Spearman’s correlation analysis, and the results were visualized using a Seaborn (v0.13.2) heatmap (Waskom, 2021). In correlation analyses, p-values < 0.05 were considered statistically significant, and the Spearman’s rho coefficients for significant metabolites are shown in the correlation heatmap.

To investigate the biological relevance of recurrent metabolomic alterations identified across group and subgroup comparisons, pathway enrichment analysis was performed using the Kyoto Encyclopedia of Genes and Genomes (KEGG) database through MetaboAnalyst. Metabolites that were recurrently identified across at least three of the six metabolite comparison groups were selected and mapped to KEGG pathways. This selection strategy was adopted to prioritize metabolite alterations demonstrating consistency across clinical comparisons and to reduce the influence of isolated subgroup-specific findings.

Enrichment statistics were calculated to identify overrepresented metabolic pathways. To reduce false-positive findings due to multiple comparisons, both Holm adjustment and false discovery rate (FDR) correction were applied. Pathways were ranked according to enrichment significance, and only pathways remaining significant after multiple testing correction were interpreted as primary findings, whereas nominally enriched pathways were considered exploratory.

## Results

### Demographic and clinical characteristics

A total of 55 patients with schizophrenia and 30 healthy controls were included in the study. Demographic and clinical characteristics of the control and patient groups are presented in Table 1, and patient-specific clinical characteristics are summarized in Table 2. The groups were comparable in terms of age and sex and did not differ significantly regarding education level, marital status, smoking status, medical illness, or anthropometric measures (all *p* > 0.05). Significant between-group differences were observed for income status and alcohol use, with patients more frequently reporting minimum wage or lower income and lower alcohol consumption than controls (Table 1).

**Table 1:** Demographic and clinical characteristics of control and patient groups

Variable	Groups		*p*-value
	Control (*n* = 30)	Patient (*n* = 55)	
Age (years)	44.93 ± 8.16	46.22 ± 8.21	0.642
Gender (female)	5 (16.7%)	14 (25.5%)	0.511
Education			
High school	7 (23.3%)	13 (23.6%)	0.231
Primary school	20 (66.7%)	41 (74.5%)	0.231
University	3 (10.0%)	1 (1.8%)	0.231
Marital status			
Divorced or widowed	5 (16.7%)	9 (16.4%)	0.637
Married	8 (26.7%)	10 (18.2%)	0.637
Single	17 (56.7%)	36 (65.5%)	0.637
Income			
above minimum wage	14 (46.7%)	9 (16.4%)	**0.006**
minimum wage or below	16 (53.3%)	46 (83.6%)	**0.006**
Cigarette (yes)	15 (50.0%)	37 (67.3%)	0.184
Alcohol use (yes)	8 (26.7%)	4 (7.3%)	**0.033**
Medical illness (yes)	8 (26.7%)	14 (25.5%)	1.000
Height (cm)	172.33 ± 7.56	168.98 ± 9.29	0.094
Weight (kg)	79.80 ± 11.34	81.65 ± 16.68	0.744
BMI (kg/m^2^)	26.93 ± 4.01	28.60 ± 5.49	0.201

Continuous variables are reported as mean ± standard deviation. Between-group comparisons were Categorical variables are presented as frequencies (percentages). Statistical significant *p*-values are shown in bold

**Table 2:** Demographic and clinical characteristics of the patient group

Variable	Patients (*n* = 55)
Age at onset of illness (years)	24.13 ± 7.79
Duration of illness (years)	22.00 [17.00–28.00]
Number of hospitalizations	4.00 [2.00–11.00]
Current treatment: antipsychotic + mood stabilizer	12 (21.8%)
Adherence to treatment	
Good	39 (70.9%)
Moderate	11 (20.0%)
Poor	5 (9.1%)
Family history of schizophrenia (yes)	17 (30.9%)
Family history of non-schizophrenic mental illness (yes)	6 (10.9%)
Suicidal attempt (yes)	19 (34.5%)
CGI-S_group (5–6)	18 (32.7%)

Continuous variables are expressed as mean ± SD and median [1st −3rd quartiles]. Categorical variables are presented as frequencies (percentages). Patients were categorized based on Clinical Global Impression–Severity (CGI-S) scores into two groups: CGI-S 3–4 (mild-to-moderate symptom severity; CGI-S scores 3–4) and CGI-S 5–6 (marked-to-severe symptom severity; CGI-S scores 5–6)

Within the schizophrenia group, the mean age at illness onset was 24.13 ± 7.79 years, median illness duration was 22.00 [17.00–28.00] years, and median number of hospitalizations was 4.00 [2.00–11.00]. Most patients showed good treatment adherence (70.9%), 21.8% received combined antipsychotic and mood stabilizer treatment, and 34.5% had a history of suicide attempts (Table 2).

Urinary creatinine concentrations did not differ significantly between groups (median [IQR]: 116.00 [100.00–137.40] mg/dL in patients vs. 119.50 [95.20–135.80] mg/dL in controls; *p* = 0.126).

### Differential urinary organic acid concentrations between patients and controls

Comparisons of urinary organic acid concentrations between patients with schizophrenia and healthy controls revealed widespread alterations in urinary metabolomic profiles. Differential metabolites identified after Benjamini–Hochberg false discovery rate (FDR) correction are summarized in Table 3, while detailed metabolite distributions are presented in Supplementary Figure S1. A total of 40 metabolites remained significantly different between groups after FDR adjustment.

**Table 3:** Differentially abundant metabolites between patient and control groups

Metabolite	Control (Median [Q1-Q3])	Patient (Median [Q1-Q3])	log2 (FC)	Up/Down	FDR
2-Hydroxy-3-Methylpentanoic acid	0.04 [0.02–0.08]	0.37 [0.14–0.57]	3.13	Up	0.00000
Alpha-Ketoglutaric acid	67.62 [55.44–91.05]	612.29 [486.53–961.99]	3.18	Up	0.00000
Citric acid	104.94 [88.98–159.24]	2268.83 [1420.80–4069.47]	4.43	Up	0.00000
Glutaric acid	0.92 [0.50–1.26]	2.23 [1.71–4.41]	1.28	Up	0.00000
Glycolic acid	37.69 [23.56–74.26]	2021.97 [1675.80–2511.64]	5.75	Up	0.00000
Homogentisic acid	0.30 [0.16–0.47]	9.16 [5.46–14.23]	4.91	Up	0.00000
Lactic acid	39.23 [27.82–67.73]	141.93 [83.06–229.62]	1.86	Up	0.00000
Adipic acid	3.16 [2.07–5.15]	10.17 [6.58–16.34]	1.68	Up	0.00000
Malic acid	2.99 [2.31–4.37]	267.75 [172.49–491.64]	6.49	Up	0.00000
Methylmalonic acid	5.61 [2.63–8.11]	30.02 [20.65–62.95]	2.42	Up	0.00000
Orotic acid	2.23 [1.22–3.03]	36.18 [21.36–52.09]	4.02	Up	0.00000
Oxoproline (Pyroglutamic acid)	65.16 [55.76–94.40]	195.92 [173.36–235.37]	1.59	Up	0.00000
Suberic acid	6.46 [4.80–7.88]	1.79 [0.93–4.19]	−1.85	Down	0.00000
Suberylglycine	0.07 [0.04–0.15]	0.56 [0.25–1.32]	3.06	Up	0.00000
Succinic acid	12.58 [7.81–23.84]	33.78 [22.67–56.38]	1.42	Up	0.00000
Malonic acid	0.57 [0.26–0.91]	24.95 [20.29–33.48]	5.45	Up	0.00000
4-Methyl-2-Oxovaleric acid	0.29 [0.17–0.67]	15.27 [6.87–26.03]	5.73	Up	0.00000
p-Hydroxyphenyllactic acid	0.78 [0.54–1.59]	4.96 [2.99–7.41]	2.67	Up	0.00000
2-Hydroxyglutaric acid	8.54 [7.02–11.51]	35.82 [24.20–53.58]	2.07	Up	0.00000
3-Methyl − 2-Oxovaleric acid	2.55 [0.95–4.78]	9.63 [5.85–16.20]	1.92	Up	0.00000
2-Hydroxyisocaproic acid (2-Hydroxy-4-methylpentanoic acid)	0.02 [0.00–0.06]	0.20 [0.09–0.46]	3.30	Up	0.00000
3-Hydroxypropanoic acid	9.87 [7.46–15.41]	35.80 [21.65–55.84]	1.86	Up	0.00000
3-Hydroxyisovaleric acid	23.30 [14.68–35.58]	59.66 [40.65–89.19]	1.36	Up	0.00000
3-Hydroxyisobutyric acid	79.60 [41.70–118.52]	6.28 [4.41–8.90]	−3.66	Down	0.00000
2-Hydroxyphenylacetic acid	3.00 [2.50–3.81]	10.59 [8.21–16.79]	1.82	Up	0.00000
3-Methylglutaric acid	1.57 [1.17–2.31]	7.20 [5.05–8.96]	2.19	Up	0.00000
2-Hydroxyisovaleric acid	0.27 [0.14–0.59]	0.98 [0.40–2.31]	1.85	Up	0.00004
Ethylmalonic acid	10.25 [6.26–18.82]	20.36 [15.24–29.77]	0.99	Up	0.00005
Propionyl glycine	0.01 [0.00–0.02]	0.05 [0.02–0.11]	2.48	Up	0.00006
3-Hydroxypentanoic acid	0.11 [0.07–0.21]	0.34 [0.15–0.54]	1.58	Up	0.00006
3-Methylglutaconic acid	24.32 [19.01–33.54]	39.93 [29.20–54.51]	0.72	Up	0.00047
Glutaconic acid	0.27 [0.14–0.45]	0.55 [0.30–1.26]	1.04	Up	0.00049
N-Acetylaspartic acid	21.82 [17.34–31.58]	38.20 [22.69–47.50]	0.81	Up	0.00049
2-Oxoadipic acid	4.02 [2.77–7.83]	8.31 [4.94–11.32]	1.05	Up	0.00050
Tiglylglycine	1.56 [0.95–3.11]	8.81 [2.32–13.09]	2.50	Up	0.00055
3-Hydroxybutyric acid	1.72 [0.76–2.83]	3.09 [2.28–6.61]	0.85	Up	0.00084
N-isovaleryl glycine	2.43 [1.26–3.22]	4.32 [2.73–5.93]	0.83	Up	0.00090
3-Methylcrotonylglycine	0.71 [0.44–1.16]	1.30 [0.73–2.41]	0.87	Up	0.00135
3-Hydroxyglutaric acid	12.88 [9.05–17.69]	23.68 [11.77–38.16]	0.88	Up	0.00343
N-Acetyltyrosine	0.90 [0.69–1.19]	0.44 [0.26–1.33]	−1.03	Down	0.01246

Metabolite levels were compared between patient and control groups using the Mann–Whitney U test. Metabolites with false discovery rate (FDR)-adjusted *p* values < 0.05 were considered statistically significant and included in the table. Log2 fold change (log2FC) represents the magnitude of change between groups, while the Up/Down direction indicates the overall trend of metabolite abundance in the patient group relative to controls

Most altered metabolites demonstrated higher urinary concentrations in the schizophrenia group relative to healthy controls, whereas a smaller subset showed reduced concentrations. Increased metabolites included compounds spanning multiple metabolic classes, whereas selected metabolites demonstrated lower concentrations in patients.

### Clinical subgroup analyses

Subgroup comparisons revealed a consistent pattern of metabolic differences, with several organic acids showing notable alterations in the schizophrenia group. These metabolites were carried forward into pathway analysis to identify converging biological themes. Detailed subgroup findings are summarized in Table 4, and corresponding metabolite distributions are presented in Supplementary Figure S2.

**Table 4:** Differentially abundant metabolites according to clinical characteristics in the patient group

4. 1. Clinical characteristics: CGI-S (5–6 vs. 3–4)					
Metabolite	CGI-S 3–4 (Median [Q1-Q3])	CGI-S 5–6 (Median [Q1-Q3])	log2 (FC)	Up/Down	*p*-value
Malonic acid	28.21 [22.76–35.99]	20.90 [18.12–28.02]	−0.43	Down	0.03506
3-Hydroxypropanoic acid	39.67 [25.51–64.76]	25.04 [15.84–44.92]	−0.66	Down	0.05163
3-Hydroxyisovaleric acid	55.19 [38.99–79.42]	72.45 [55.45–124.76]	0.39	Up	0.05844
N-isovaleryl glycine	3.76 [2.30–5.71]	4.84 [4.33–7.09]	0.37	Up	0.05844
3-Methyl − 2-Oxovaleric acid	8.45 [5.50–15.62]	13.95 [7.60–24.30]	0.72	Up	0.07143
2-Hydroxyisocaproic acid (2-Hydroxy-4-methylpentanoic acid)	0.18 [0.09–0.36]	0.34 [0.10–1.02]	0.94	Up	0.07143
3-Hydroxypentanoic acid	0.39 [0.22–0.55]	0.21 [0.11–0.37]	−0.87	Down	0.07726
Suberylglycine	0.52 [0.18–0.96]	0.94 [0.50–2.04]	0.87	Up	0.09707
3-Hydroxyglutaric acid	24.62 [12.86–38.24]	17.87 [6.06–31.25]	−0.46	Down	0.09889

4. 2. Clinical characteristics: Adherence to Treatment (Good vs. Poor-Moderate)					
Metabolite	Poor-Moderate (Median [Q1-Q3])	Good (Median [Q1-Q3])	log2 (FC)	Up/Down	*p*-value
Citric acid	1420.80 [1259.99–2630.86]	2942.63 [1877.52–4702.60]	1.05	Up	0.0221
3-Hydroxyisobutyric acid	4.76 [4.28–5.95]	7.34 [5.40–9.70]	0.62	Up	0.0221
3-Hydroxypentanoic acid	0.21 [0.10–0.32]	0.39 [0.22–0.80]	0.91	Up	0.0232
Adipic acid	8.39 [4.64–10.66]	11.83 [7.11–18.78]	0.50	Up	0.0268
Lactic acid	102.87 [74.72–164.60]	154.95 [97.64–283.06]	0.59	Up	0.03385
Glutaric acid	1.96 [1.66–2.35]	3.04 [1.86–5.01]	0.64	Up	0.03385
p-Hydroxyphenyllactic acid	4.30 [1.82–4.99]	5.58 [3.64–9.23]	0.38	Up	0.03881
2-Hydroxy-3-Methylpentanoic acid	0.15 [0.12–0.44]	0.41 [0.18–0.72]	1.43	Up	0.04244
Succinic acid	27.81 [17.64–38.19]	37.77 [23.06–67.70]	0.44	Up	0.04636
3-Hydroxybutyric acid	2.45 [1.95–3.03]	4.39 [2.51–11.23]	0.84	Up	0.05511
Succunylacetone	0.04 [0.01–0.06]	0.06 [0.03–0.12]	0.78	Up	0.05998

4. 3. Clinical characteristics: Family History of Schizophrenia (Yes vs. No)					
Metabolite	No (Median [Q1-Q3])	Yes (Median [Q1-Q3])	log2 (FC)	Up/Down	*p*-value
Succunylacetone	0.07 [0.04–0.14]	0.03 [0.01–0.04]	−1.09	Down	0.00274
Propionyl glycine	0.06 [0.03–0.13]	0.02 [0.00–0.05]	−1.40	Down	0.00849
Methylmalonic acid	42.07 [24.35–74.19]	21.77 [15.36–30.45]	−0.95	Down	0.00896
Glutaconic acid	0.67 [0.40–1.61]	0.37 [0.22–0.63]	−0.84	Down	0.0143
3-Hydroxypentanoic acid	0.39 [0.22–0.73]	0.19 [0.07–0.39]	−1.02	Down	0.02123
3-Hydroxyglutaric acid	20.57 [11.05–33.10]	32.24 [22.81–40.97]	0.65	Up	0.02819
Orotic acid	37.79 [25.32–58.28]	23.57 [18.21–39.52]	−0.68	Down	0.05701
2-Hydroxyphenylacetic acid	11.93 [9.13–18.72]	9.43 [7.72–11.31]	−0.34	Down	0.05942
2-Hydroxy-3-Methylpentanoic acid	0.40 [0.17–0.61]	0.14 [0.07–0.54]	−1.55	Down	0.06719
3-Methylglutaconic acid	34.65 [28.17–49.65]	46.95 [37.16–54.53]	0.44	Up	0.08523
3-Methyl − 2-Oxovaleric acid	11.08 [6.08–22.40]	7.83 [5.89–11.00]	−0.50	Down	0.08858

4. 4. Clinical characteristics: Suicidal Attempt (Yes vs. No)					
Metabolite	No (Median [Q1-Q3])	Yes (Median [Q1-Q3])	log2 (FC)	Up/Down	*p*-value
p-Hydroxyphenyllactic acid	5.38 [4.22–9.71]	3.91 [2.50–5.62]	−0.46	Down	0.06964

4. 5. Clinical characteristics: Current Treatment (AP + MS vs. AP)					
Metabolite	AP (Median [Q1-Q3])	AP + MS (Median [Q1-Q3])	log2 (FC)	Up/Down	*p*-value
3-Methylcrotonylglycine	1.14 [0.66–2.14]	2.86 [1.73–7.78]	1.33	Up	0.00143
2-Hydroxyglutaric acid	31.39 [21.72–47.79]	52.44 [39.17–130.53]	0.74	Up	0.00652
Glutaric acid	2.19 [1.65–3.23]	4.71 [2.95–11.94]	1.10	Up	0.01054
Succinic acid	32.53 [20.28–42.47]	65.70 [30.58–101.70]	1.01	Up	0.01118
3-Hydroxypropanoic acid	39.67 [24.51–65.02]	20.17 [14.56–31.96]	−0.98	Down	0.01329
N-isovaleryl glycine	3.84 [2.35–5.02]	5.77 [4.37–10.06]	0.59	Up	0.01406
Adipic acid	9.02 [6.28–13.44]	19.48 [9.88–33.20]	1.11	Up	0.02703
N-Acetyltyrosine	0.40 [0.24–0.90]	1.24 [0.44–3.22]	1.64	Up	0.03321
Malic acid	249.06 [170.10–413.08]	498.84 [286.73–751.23]	1.00	Up	0.03493
Suberylglycine	0.52 [0.19–0.96]	1.37 [0.52–2.60]	1.40	Up	0.03493
2-Oxoadipic acid	7.43 [4.72–11.05]	10.08 [8.57–13.40]	0.44	Up	0.07794
3-Hydroxyisovaleric acid	55.78 [39.98–77.42]	107.15 [51.70–166.17]	0.94	Up	0.08883

Metabolite levels were compared between clinical subgroups within the patient cohort using the Mann–Whitney U test. Five clinical comparisons were evaluated: CGI-S score (5–6 vs. 3–4), adherence to treatment (good vs. poor-moderate), family history of schizophrenia (yes vs. no), suicidal attempt history (yes vs. no), and current treatment (antipsychotic + mood stabilizer [AP + MS] vs. antipsychotic only [AP]). Metabolites with *p* values < 0.10 were considered trend-level findings and included in the table. Log2 fold change (log2FC) represents the magnitude of change between comparison groups, while the Up/Down direction indicates the relative trend of metabolite abundance according to the first group listed in each comparison

According to symptom severity (CGI-S), malonic acid demonstrated lower levels in patients with marked-to-severe symptom severity, while 3-hydroxypropanoic acid showed a trend toward lower levels. In contrast, 3-hydroxyisovaleric acid and N-isovaleryl glycine exhibited nominally higher levels in patients with marked-to-severe symptom severity. Similarly, subgroup analyses according to treatment adherence identified higher concentrations of citric acid, lactic acid, succinic acid, and 3-hydroxypentanoic acid in patients with good treatment adherence, with additional metabolites showing trend-level differences across adherence categories.

Family history analyses revealed one of the most distinct subgroup-level metabolic profiles. Propionyl glycine, methylmalonic acid, and succinylacetone showed lower concentrations in patients with a positive family history of schizophrenia, whereas 3-hydroxyglutaric acid exhibited nominally higher levels.

Only limited metabolic alterations were observed according to suicidal attempt history, with p-hydroxyphenyllactic acid showing a tendency toward lower concentrations. Additional exploratory analyses according to current treatment status (antipsychotic monotherapy vs. combined antipsychotic plus mood stabilizer treatment) identified differences in selected metabolites, including succinic acid, 3-hydroxypropanoic acid, malic acid, and 2-hydroxyglutaric acid. Several metabolites exhibited nominally altered levels across treatment categories.

### Principal component analysis

**Fig. 1:**
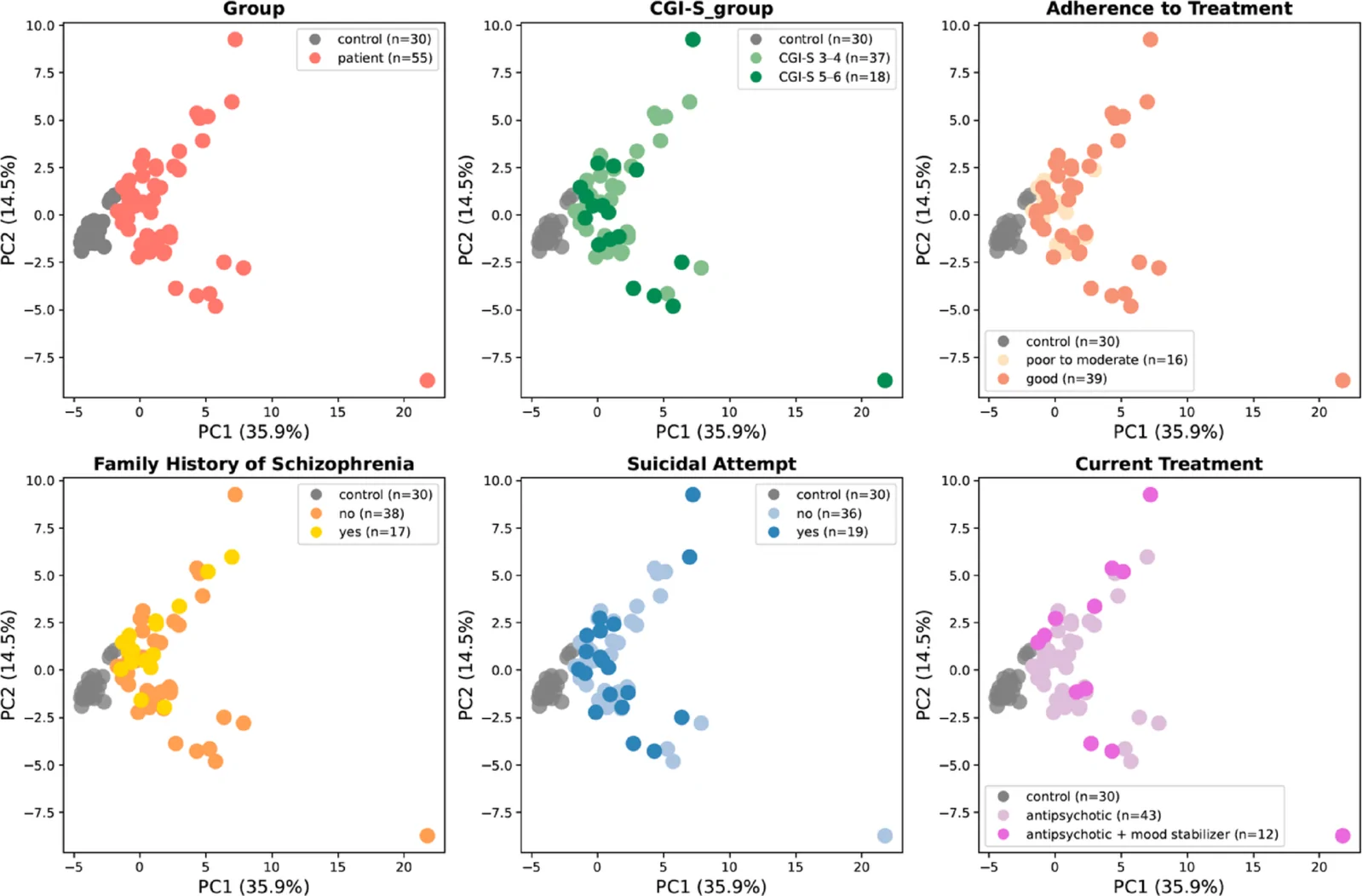
Principal component analysis (PCA) of urinary organic acid profiles across patient and clinical subgroup comparisons. PCA was performed using standardized urinary organic acid concentrations. The first two principal components explained 35.9% (PC1) and 14.5% (PC2) of the total variance. The upper-left panel presents the distribution of patients with schizophrenia and healthy controls. Additional panels illustrate exploratory subgroup visualizations according to symptom severity (Clinical Global Impression–Severity; CGI-S), treatment adherence (good vs. poor-to-moderate), family history of schizophrenia (yes vs. no), suicidal attempt history (yes vs. no), and current treatment status (antipsychotic monotherapy [AP] vs. combined antipsychotic plus mood stabilizer treatment [AP + MS])

Principal component analysis (PCA) was performed using standardized urinary organic acid concentrations to evaluate global metabolomic patterns. The first two principal components explained 35.9% and 14.5% of the total variance, respectively. As shown in Fig. 1, patients with schizophrenia demonstrated partial separation from healthy controls, indicating differences in overall urinary metabolomic profiles.

Exploratory PCA visualizations according to clinical subgroup characteristics, including symptom severity (CGI-S), treatment adherence, family history of schizophrenia, suicidal attempt history, and current treatment status, showed substantial overlap across subgroup categories without evidence of distinct clustering.

### Correlation analyses

**Fig. 2:**
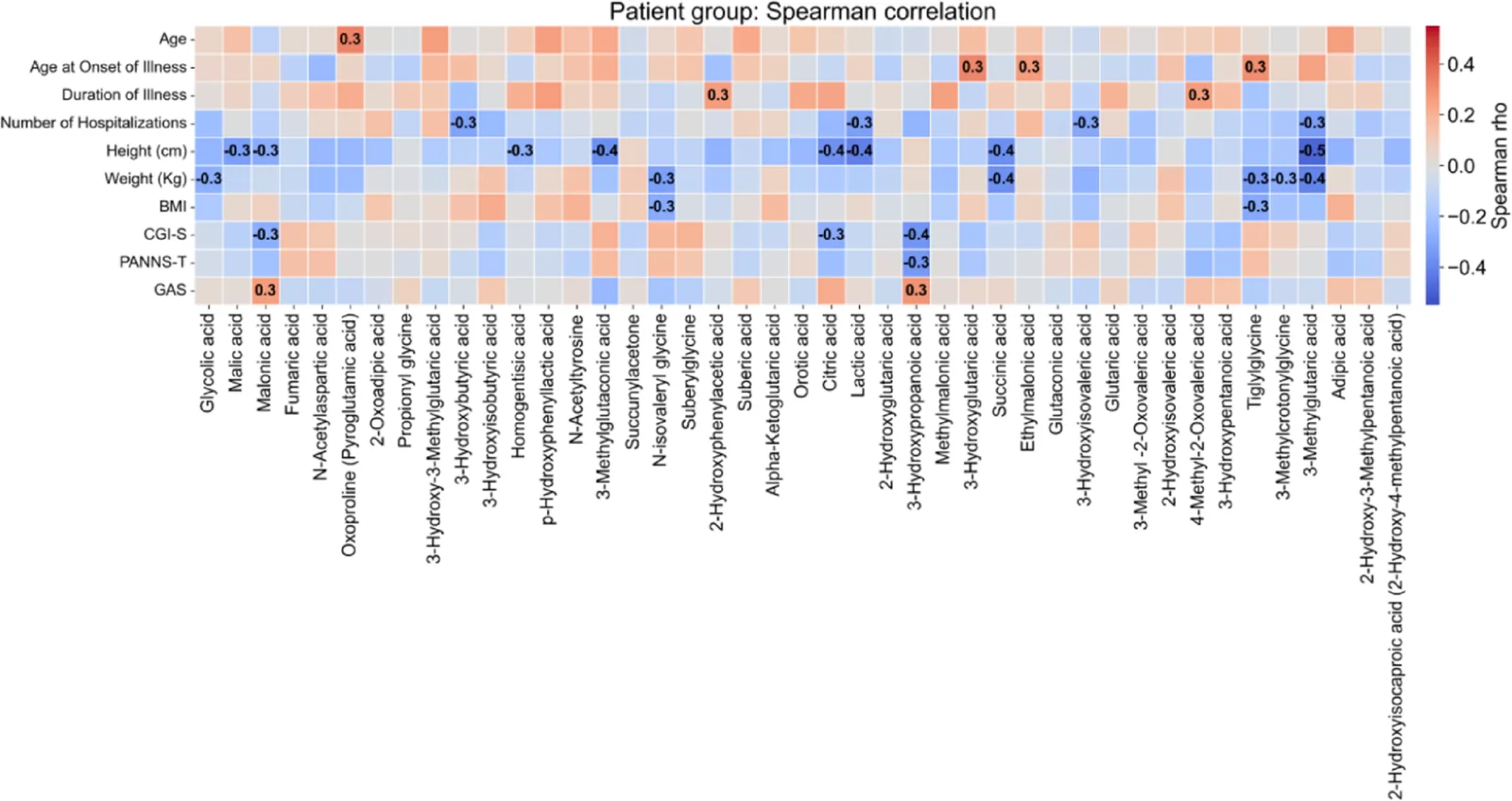
Spearman correlation heatmap between clinical characteristics and urinary organic acid concentrations in patients with schizophrenia. Heatmap illustrating Spearman correlation coefficients (ρ) between clinical variables and normalized urinary organic acid concentrations in the patient group. Positive correlations are shown in red and negative correlations in blue. Only statistically significant correlations are annotated. Clinical variables included age, age at onset of illness, duration of illness, number of hospitalizations, anthropometric measures, symptom severity (CGI-S), psychopathology severity (PANSS-T), and global functioning (GAS)

Associations between clinical variables and urinary organic acid concentrations were evaluated using Spearman correlation analysis and are summarized in Fig. 2. Overall, correlation coefficients were predominantly within the weak-to-moderate range.

Positive correlations were observed between age at onset of illness and selected metabolites, while duration of illness demonstrated positive associations with several urinary organic acids. In contrast, the number of hospitalizations showed predominantly inverse correlations with selected metabolites.

Clinical severity measures, including CGI-S and PANSS total scores, exhibited mostly weak negative correlations with several metabolites, whereas functional status (GAS) demonstrated limited positive associations. Anthropometric variables, particularly height and weight, also showed multiple inverse correlations across selected metabolites.

Overall, correlation analyses demonstrated distributed but generally weak-to-moderate associations between clinical variables and urinary metabolite concentrations.

### Pathway enrichment analysis

To identify shared biological processes underlying recurrent metabolomic alterations, pathway enrichment analysis was performed using metabolites identified across at least three clinical comparison groups.

Pathway enrichment analysis revealed propanoate metabolism as the only pathway that remained statistically significant after multiple testing correction (raw *p* = 0.000596, Holm-adjusted *p* = 0.0483, FDR = 0.0483). Additional pathways showing nominal enrichment included valine, leucine and isoleucine biosynthesis, butanoate metabolism, citrate cycle (TCA cycle), beta-alanine metabolism, alanine, aspartate and glutamate metabolism, and valine, leucine and isoleucine degradation, although these pathways did not remain significant after correction (Table 5).

**Table 5:** KEGG pathway enrichment analysis of metabolites shared across three or more clinical comparisons

KEGG pathway	Total	Hits	Raw *p*-value	Holm *p*-value	FDR
Propanoate metabolism	22	2	0.000596	0.0483	0.0483
Valine, leucine and isoleucine biosynthesis	8	1	0.0157	1	0.637
Butanoate metabolism	15	1	0.0294	1	0.663
Citrate cycle (TCA cycle)	20	1	0.039	1	0.663
beta-Alanine metabolism	21	1	0.0409	1	0.663
Alanine, aspartate and glutamate metabolism	28	1	0.0543	1	0.733
Valine, leucine and isoleucine degradation	40	1	0.077	1	0.891

KEGG pathway enrichment analysis was performed using MetaboAnalyst for metabolites that were commonly identified in at least three of the six metabolite comparison groups. Pathways associated with the shared metabolites are presented along with enrichment statistics and adjusted significance values

Collectively, these findings identified propanoate metabolism as the only pathway that remained significant after multiple testing correction.

## Discussion

Schizophrenia remains a complex and debilitating psychiatric disorder, and its underlying neurobiological mechanisms are not yet fully understood. Beyond conventional neurochemical and genetic models, increasing evidence suggests that metabolic disturbances may contribute to the biological complexity of schizophrenia and may provide additional insight into disease heterogeneity and systemic biological alterations (Berk et al., 2025; Cai et al., 2012; Cao et al., 2020; Henkel et al., 2022).

In the present study, we identified widespread alterations in urinary organic acid profiles in patients with schizophrenia together with subgroup-related metabolic variation and pathway-level enrichment findings. Notably, propanoate metabolism was the only pathway that remained statistically significant after multiple testing correction, whereas several additional pathways demonstrated nominal enrichment. These findings suggest that urinary metabolomic alterations associated with schizophrenia may involve broader metabolic reorganization across interconnected biological processes rather than isolated abnormalities within a single pathway.

At the same time, the broad pattern of urinary metabolite alterations observed in our study should be interpreted cautiously and should not be assumed to represent uniform activation of metabolic pathways. Alternative explanations, including assumptions related to creatinine normalization, treatment-related metabolic effects, altered systemic metabolism, dietary influences, microbiome-related variability, and differences in renal handling should also be considered. Since urinary metabolites reflect systemic physiology rather than brain-specific metabolism alone, the present findings are more appropriately interpreted as indirect indicators of broader metabolic changes associated with schizophrenia rather than direct evidence of mitochondrial dysfunction or brain-specific metabolic abnormalities.

Although butanoate metabolism did not remain statistically significant after correction for multiple comparisons, it was among the pathways showing nominal enrichment and may provide contextual information for interpreting broader metabolic alterations. Butanoate is a short-chain fatty acid generated through gut microbial metabolism and has been implicated in biological processes relevant to psychiatric disorders, including regulation of blood–brain barrier integrity, neuroimmune signaling, and energy homeostasis (Adebayo et al., 2025; Chakraborty et al., 2024; Liu et al., 2018; Stiernborg et al., 2024). Evidence from schizophrenia research further suggests that alterations in butyrate-producing microbiota may contribute to systemic metabolic dysregulation and may influence clinical outcomes (Kamath et al., 2025; Zheng et al., 2019).

In the present study, altered urinary concentrations of metabolites including lactate and succinic acid were observed in patients with schizophrenia. Since these metabolites participate in interconnected pathways involving short-chain fatty acid metabolism and intermediary metabolism, the observed findings may be compatible with broader metabolic alterations extending beyond a single pathway. However, because butanoate metabolism did not remain significant after multiple testing correction and urinary metabolites reflect systemic rather than brain-specific physiology, these observations should be interpreted as exploratory rather than mechanistic evidence of altered gut microbiota activity.

One of the subgroup findings in the present study was the observation of lower urinary malonic acid concentrations in patients with higher symptom severity as measured by CGI-S. Malonic acid is closely linked to fatty acid metabolism through its relationship with malonyl-CoA, a central intermediate generated by acetyl-CoA carboxylase (ACC), which regulates long-chain fatty acid synthesis and influences mitochondrial fatty acid oxidation through inhibition of carnitine palmitoyltransferase I (CPT1) (de Leon et al., 2008; Albaugh et al., 2012; Li et al., 2022).

Alterations in fatty acid metabolism have previously been associated with metabolic disturbances observed in schizophrenia and may also be influenced by pharmacological treatment. Antipsychotic exposure has been linked to dyslipidemia and broader metabolic changes, including alterations in lipid regulation and energy homeostasis (Li et al., 2020; Yan et al., 2013). Since all patients in our study were receiving psychotropic treatment and treatment-related subgroup analyses also demonstrated selected metabolite differences, the observed association between lower malonic acid concentrations and greater symptom severity should be interpreted cautiously. Rather than reflecting a disease-specific mechanism, these findings may represent the combined influence of illness severity and treatment-related metabolic effects. Further studies involving drug-naïve cohorts and longitudinal designs are needed to clarify the biological relevance of malonic acid alterations in schizophrenia.

One of the most distinct subgroup-related findings in the present study was the observation of differential metabolite profiles according to family history of schizophrenia. Specifically, patients with a positive family history exhibited lower urinary concentrations of propionyl glycine, methylmalonic acid, and succinylacetone, whereas 3-hydroxyglutaric acid showed higher levels. Propionyl glycine and methylmalonic acid are closely associated with propanoate metabolism and branched-chain amino acid (BCAA) catabolism and are commonly considered indicators of intermediary metabolic activity (Deodato et al., 2006; Head et al., 2022; Longo et al., 2022).

The biological relevance of these pathways is supported by evidence that severe disruptions in propanoate metabolism lead to inherited metabolic disorders such as propionic and methylmalonic acidemia (Deodato et al., 2006; Huemer et al., 2017). Although our findings do not suggest comparable metabolic defects, they may indicate more subtle alterations in metabolic regulation. Since family history is generally considered a proxy for genetic vulnerability, the observed metabolomic differences raise the possibility that subgroup-related metabolic variation in schizophrenia may partly reflect inherited biological heterogeneity rather than symptom severity alone.

Because BCAA metabolism contributes to intermediary metabolic processes and supports broader cellular energy regulation (Mochel, 2017; Ott et al., 2005; Sonnewald, 2014), these findings may suggest that familial vulnerability in schizophrenia is accompanied by alterations extending beyond clinical presentation alone. However, given the exploratory subgroup design and modest sample size, these observations should be interpreted cautiously and require replication in independent cohorts.

In addition to subgroup-specific metabolite findings, principal component analysis demonstrated partial separation between patients with schizophrenia and healthy controls, supporting the presence of global differences in urinary metabolomic profiles. However, PCA visualizations across clinical subgroups showed substantial overlap and did not support the presence of clearly separable metabolomic subtypes. Consistent with this observation, correlation analyses revealed predominantly weak-to-moderate associations between clinical variables and urinary metabolite concentrations. Together, these findings suggest that metabolic variation in schizophrenia may be distributed across multiple biological dimensions rather than being explained by individual clinical characteristics alone.

Additional exploratory analyses according to current treatment status identified differences across selected urinary metabolites, suggesting that treatment-related factors may contribute to part of the observed metabolomic variability. Patients receiving combined antipsychotic and mood stabilizer treatment demonstrated differences in metabolites including succinic acid, 3-hydroxypropanoic acid, malic acid, and 2-hydroxyglutaric acid compared with patients receiving antipsychotic monotherapy. These findings are consistent with previous evidence indicating that psychotropic medications may influence systemic metabolism, including pathways related to lipid regulation, energy metabolism, and mitochondrial function.

At the same time, treatment-related differences should be interpreted cautiously. Since all patients in the present study were receiving pharmacological treatment and treatment allocation was not randomized, it is not possible to distinguish medication-related effects from illness-related metabolic alterations. Therefore, the observed urinary metabolomic profile likely reflects the combined influence of schizophrenia-related biological processes and treatment-associated metabolic changes. Future studies involving drug-naïve patients and longitudinal metabolomic assessments will be important for clarifying these relationships.

Our findings suggest that schizophrenia may be associated with broad metabolic alterations involving intermediary metabolism, fatty acid–related pathways, and gut microbiota–associated metabolic processes. Among the identified pathways, propanoate metabolism remained significant after multiple testing correction, while additional pathway- and subgroup-level findings suggested distributed metabolic variation across clinical characteristics. The observed metabolomic alterations, together with the distinct subgroup profiles associated with family history, support the possibility that metabolic pathways may contribute to the biological complexity and heterogeneity of schizophrenia.

These findings highlight the potential value of metabolomic profiling in advancing our understanding of systemic metabolic alterations associated with schizophrenia. At the same time, the present results should be interpreted as exploratory and hypothesis-generating rather than mechanistic evidence of disease-specific metabolic dysfunction. Further studies are needed to clarify the biological relevance and clinical implications of these metabolic alterations.

## Limitations

This study has several limitations. First, the relatively modest sample size may limit the generalizability of the findings and should be considered particularly when interpreting subgroup analyses based on symptom severity, family history, treatment adherence, and current treatment status. Second, the cross-sectional design does not allow causal inferences regarding whether the observed metabolomic alterations reflect disease-related biological processes, treatment-associated changes, or broader systemic metabolic adaptations.

Another important limitation is that all patients were receiving antipsychotic treatment. Medication dose, treatment duration, cumulative exposure, and the effects of individual antipsychotic agents were not evaluated separately. Although additional subgroup analyses according to current treatment status were performed, treatment-related metabolic effects cannot be excluded and may have contributed to the observed metabolomic variability.

Urinary metabolite concentrations were normalized to creatinine levels. Although urinary creatinine concentrations were additionally evaluated and did not differ significantly between patients and controls, creatinine normalization may not fully account for residual variability related to renal handling, hydration status, or broader systemic metabolic factors that could influence urinary metabolite profiles.

In addition, dietary habits, physical activity, microbiota composition, and other lifestyle-related factors that may affect organic acid and short-chain fatty acid metabolism were not systematically assessed. Metabolite measurements were also based on a single urine sample and therefore may not fully capture intraindividual metabolic variability over time.

Finally, although pathway enrichment and subgroup-related metabolic differences were identified, the exploratory nature of metabolite selection and the absence of genetic, microbiome, and external validation analyses limit biological interpretation of the findings. Future longitudinal, multi-center, and multi-modal studies integrating metabolomic, genetic, and microbiome data will be important for validating and extending these observations.

## Conclusion

In conclusion, the present study provides evidence that schizophrenia is associated with broad alterations in urinary organic acid profiles, suggesting the involvement of interconnected metabolic processes extending beyond isolated biochemical pathways. Pathway enrichment analysis identified propanoate metabolism as the most robust pathway after multiple testing correction, while subgroup analyses revealed additional metabolomic variation according to clinical characteristics and family history.

Together, these findings support the possibility that metabolic alterations may contribute to the biological complexity and heterogeneity of schizophrenia. Although the present findings should be interpreted within the context of their exploratory design and require validation in larger and longitudinal cohorts, they highlight the potential value of urinary metabolomic profiling as a non-invasive approach for improving our understanding of systemic metabolic changes associated with schizophrenia.

## Supplementary Information

Below is the link to the electronic supplementary material.

Supplementary Material 1

## Data Availability

The datasets generated and/or analysed during the current study are available from the corresponding author upon reasonable request.
