# Supplementary material for: Urinary organic acid levels and their associations with clinical characteristics in patients with schizophrenia
Source: Metabolomics. 2026 Jun 16;22(4):96. doi: 10.1007/s11306-026-02479-5 (PMC13272255; doi:10.1007/s11306-026-02479-5)
Supplement: Supplementary file 1 — Supplementary Material 1 [file 11306_2026_2479_MOESM1_ESM.docx]

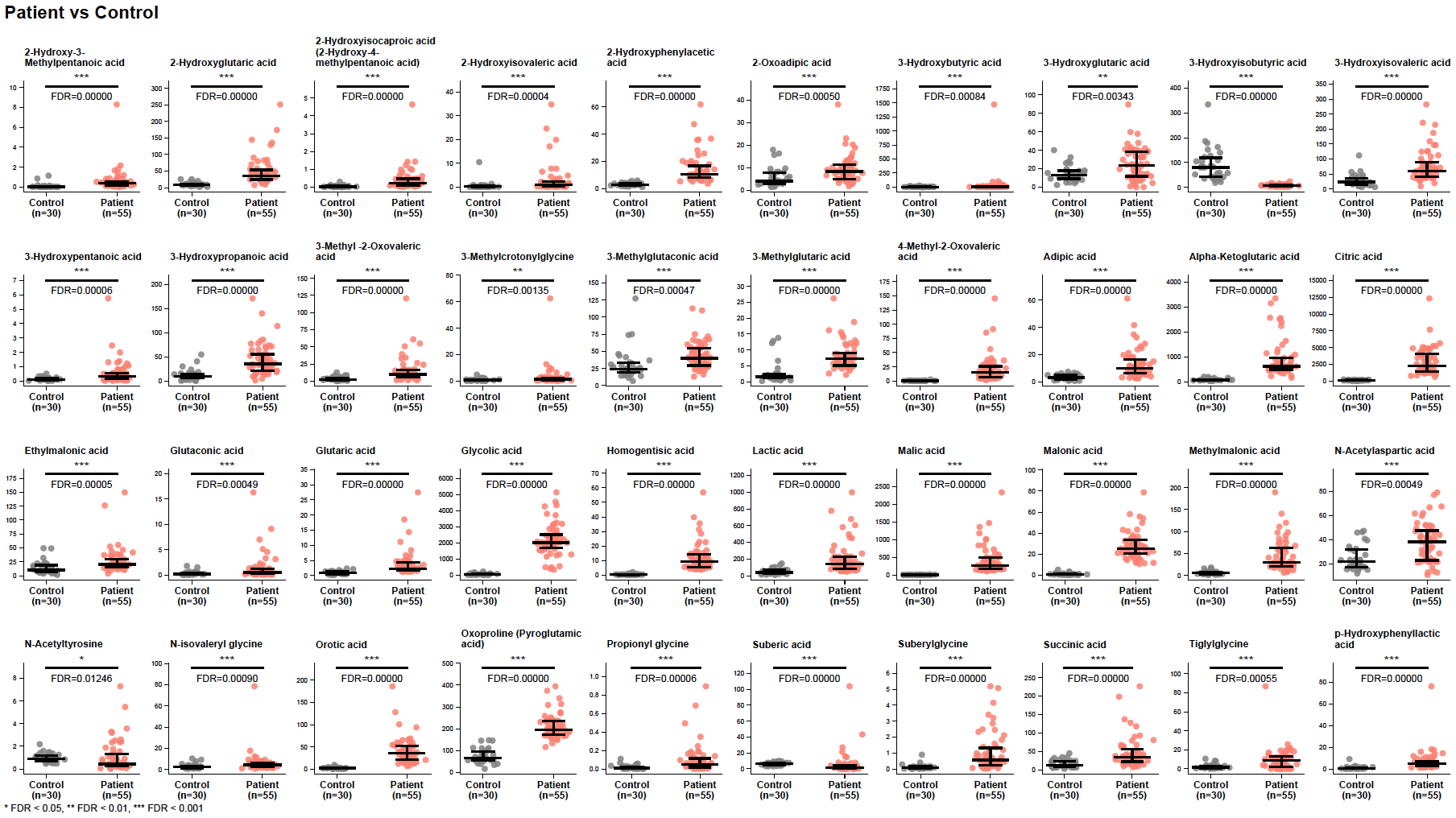


**Supplementary Figure S1. Distribution of urinary organic acid concentrations between patients with schizophrenia and healthy controls.**

Boxplots showing the distribution of urinary concentrations for all metabolites that remained significantly different between groups after Benjamini–Hochberg false discovery rate (FDR) correction. Each panel represents a single metabolite and displays individual observations together with group-level summary statistics. Metabolite concentrations are presented as normalized values (µmol/mmol creatinine). Detailed quantitative results, including fold changes and adjusted p values, are provided in Table 3.


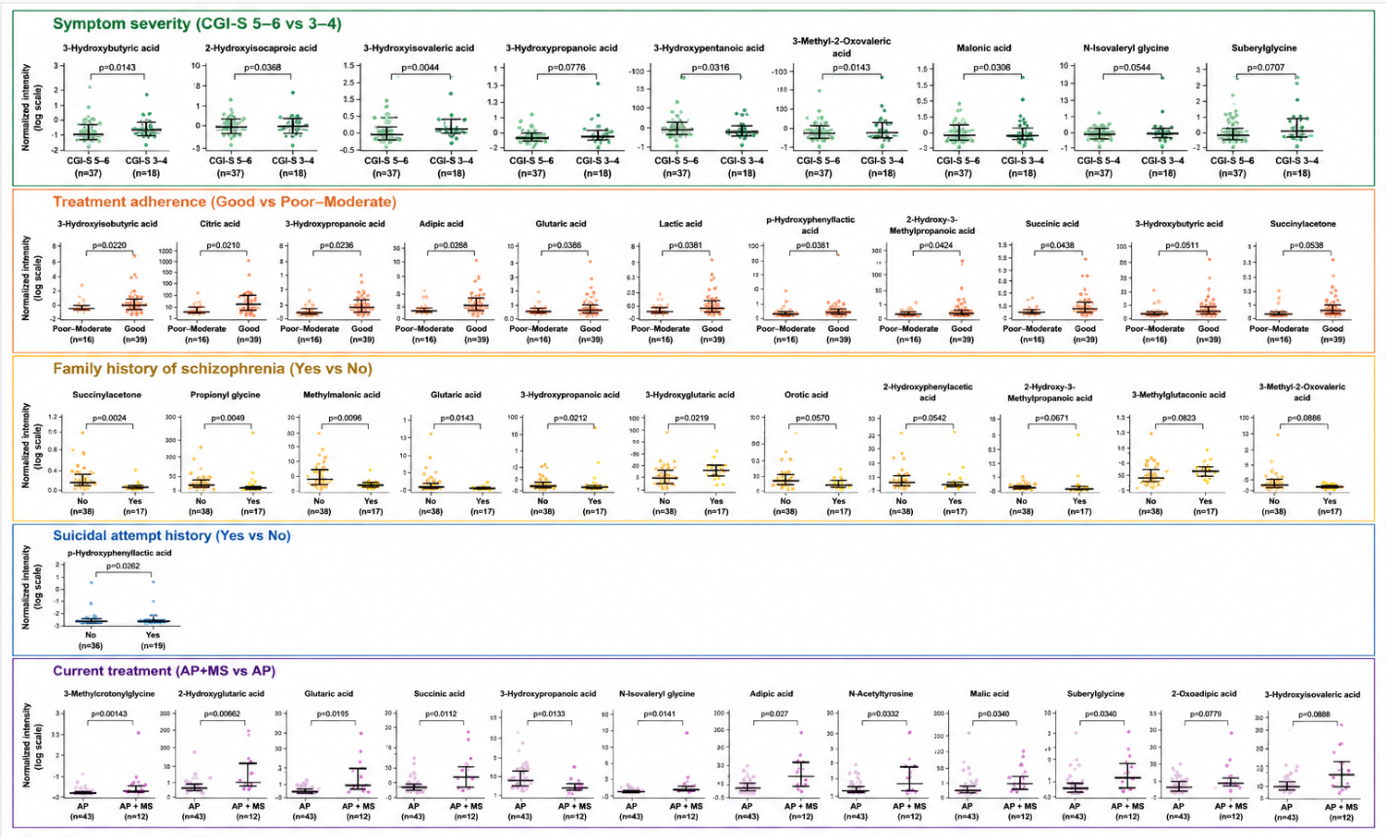


**Supplementary Figure S2. Distribution of urinary organic acid concentrations across clinical subgroup analyses in patients with schizophrenia.**

Boxplots illustrating subgroup comparisons according to symptom severity, treatment adherence, family history, suicidal attempt history, and current treatment status. Symptom severity was defined using the Clinical Global Impression–Severity scale (CGI-S), comparing patients with mild-to-moderate symptom severity (CGI-S 3–4) and marked-to-severe symptom severity (CGI-S 5–6). Treatment adherence was categorized as good versus poor-to-moderate adherence based on clinical assessment. Family history indicates the presence or absence of schizophrenia in first- or second-degree relatives. Suicidal attempt history was classified as present or absent. Current treatment status compared patients receiving antipsychotic monotherapy (AP) with those receiving combined antipsychotic plus mood stabilizer treatment (AP+MS). The figure includes metabolites demonstrating differential abundance or trend-level alterations identified in subgroup analyses. Individual points represent participant-level observations and horizontal bars indicate group-level summary statistics. Detailed quantitative results are provided in Table 4.


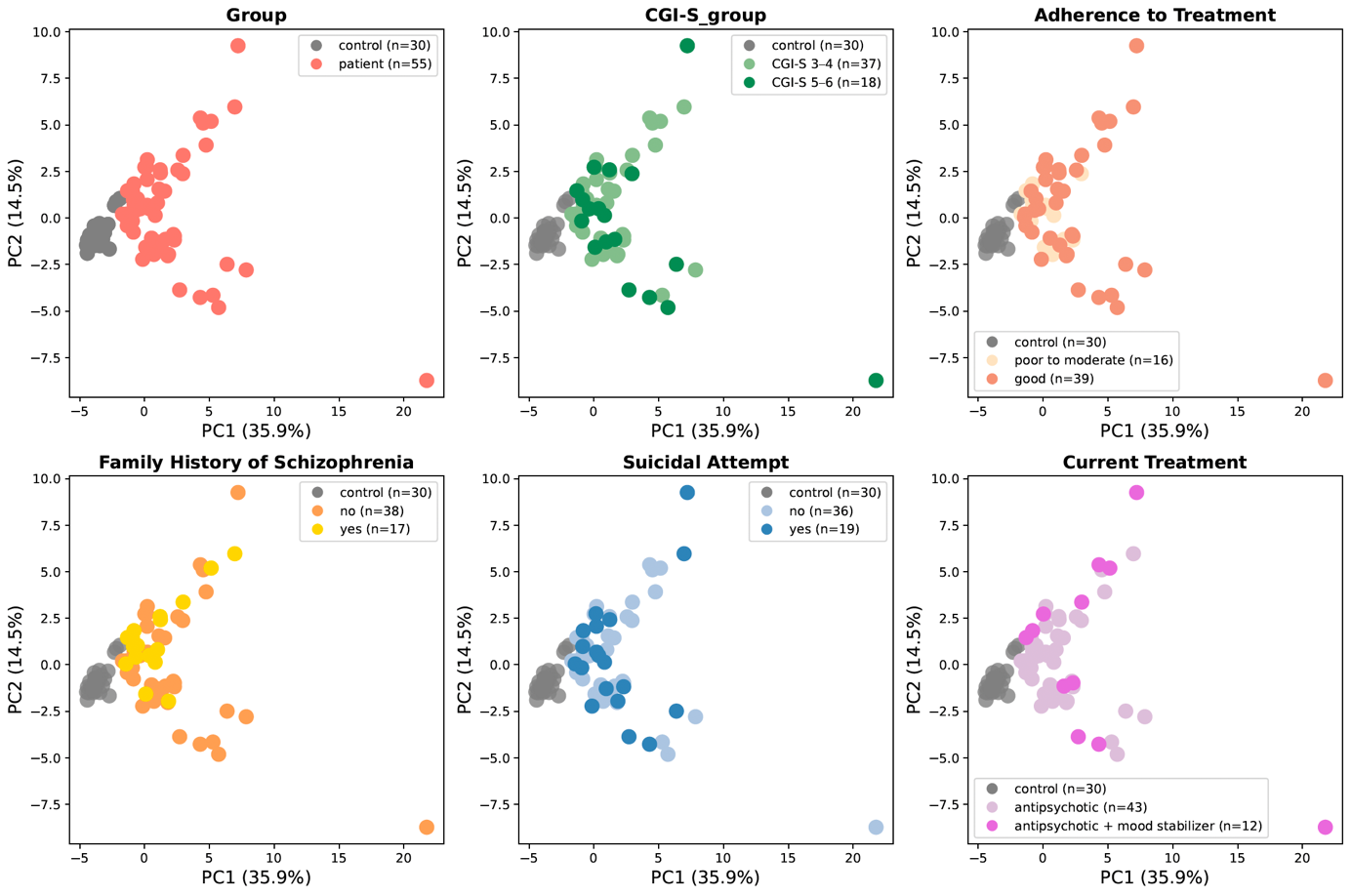


**Figure 2. Principal component analysis (PCA) of normalized urinary organic acid profiles.**

The first two principal components explained 35.9% and 14.5% of the total variance, respectively. The upper-left panel presents the comparison between patients with schizophrenia and healthy controls. Additional panels display exploratory subgroup visualizations according to symptom severity (CGI-S), treatment adherence, family history of schizophrenia, suicidal attempt history, and current treatment status.
